# Pulmonary thromboembolism with prolonged pulmonary infarction during intensive decongestion for severe heart failure: A case report

**DOI:** 10.1097/MD.0000000000051388

**Published:** 2026-10-30

**Authors:** A-Reum Roh, Jong Hyun Baek, Jong-Il Park

**Affiliations:** a Department of Internal Medicine, Yeungnam University Medical Center, Daegu, Republic of Korea; b Department of Thoracic and Cardiovascular Surgery, Yeungnam University Medical Center, Daegu, Republic of Korea; c Division of Cardiology, Department of Internal Medicine, Yeungnam University Medical Center, Daegu, Republic of Korea.

**Keywords:** decongestion, heart failure with reduced ejection fraction, pulmonary infarction, pulmonary thromboembolism

## Abstract

**Rationale:**

Pulmonary thromboembolism (PTE) may be overlooked in acute decompensated heart failure because dyspnea, hypoxemia, pleural effusion, and pulmonary infiltrates can mimic refractory congestion. Extensive PTE with pulmonary infarction during intensive decongestion in severe heart failure with reduced ejection fraction (HFrEF) is rarely described.

**Patient concerns:**

A 50-year-old woman without prior cardiovascular disease presented with several weeks of progressive dyspnea, severe orthopnea, pulmonary edema, pleural effusions, and an oxygen requirement of 5 L/min.

**Diagnoses:**

Echocardiography showed a severely dilated left ventricle and a left ventricular ejection fraction of 25%, consistent with nonischemic dilated cardiomyopathy. After a cumulative negative fluid balance of 12 L over 48 hours, hypoxemia worsened despite improved congestion. Computed tomography pulmonary angiography confirmed extensive acute PTE with pulmonary infarction.

**Interventions:**

Systemic thrombolysis with alteplase was administered, followed by unfractionated heparin and transition to a non-vitamin K antagonist oral anticoagulant. Guideline-directed medical therapy for HFrEF was initiated.

**Outcomes:**

Hemodynamics stabilized, but parenchymal recovery was delayed. Pulmonary infarction showed minimal radiographic improvement at 2 weeks, partial improvement at 1 month, and complete resolution at 2 months. Oxygen therapy was discontinued, dyspnea improved markedly, and left ventricular ejection fraction increased to 37%.

**Lessons:**

PTE should be suspected when oxygenation worsens despite effective decongestion in severe HFrEF. Pulmonary infarction may require up to 2 months for complete radiographic resolution despite prompt thrombolysis and anticoagulation.

## 1. Introduction

Heart failure with reduced ejection fraction (HFrEF) is associated with an increased risk of venous thromboembolism through reduced cardiac output, venous stasis, endothelial dysfunction, and neurohormonal activation.^[1-4]^ Pulmonary thromboembolism (PTE) may complicate the course of hospitalized patients with severe decompensated heart failure, but its diagnosis can be delayed because dyspnea, hypoxemia, tachycardia, pleural effusion, and radiographic pulmonary abnormalities overlap with the manifestations of heart failure itself.^[2,3,5]^ Intravenous diuretic therapy is central to the management of acute decompensated heart failure, but substantial intravascular volume contraction may be accompanied by hemoconcentration, particularly in patients with severely impaired cardiac output.^[6-8]^ When oxygenation worsens despite objective improvement in pulmonary congestion, clinicians should reconsider alternative or concomitant diagnoses, including PTE. We report a case of extensive acute PTE with pulmonary infarction after intensive decongestion in newly diagnosed severe HFrEF, followed by delayed but complete radiographic recovery at 2 months.

## 2. Case presentation

A 50-year-old woman with no prior cardiovascular disease presented with several weeks of progressive dyspnea, New York Heart Association class III symptoms, severe orthopnea, and an oxygen requirement of 5 L/min. On admission, she was tachycardic (130–137 beats/min) and had jugular venous distension, bilateral basal crackles, and pitting edema. Chest radiography showed bilateral pulmonary edema and pleural effusions, and electrocardiography demonstrated sinus tachycardia without ischemic changes or definite right heart strain (Fig. 1). Transthoracic echocardiography revealed a severely dilated left ventricle (end-diastolic diameter, 60 mm) with left ventricular ejection fraction (LVEF) of 25%, mild mitral regurgitation, and an estimated right ventricular systolic pressure of 42 mm Hg, without regional wall motion abnormalities. Right ventricular systolic function was also impaired, with a fractional area change of 27%. Coronary angiography demonstrated no significant luminal stenosis in the epicardial coronary arteries, supporting the diagnosis of nonischemic dilated cardiomyopathy (Video S1, Supplemental Digital Content 1). N-terminal pro–B-type natriuretic peptide was markedly elevated at 6369 pg/mL, consistent with decompensated heart failure. Cardiac troponin, thyroid-stimulating hormone, and ferritin were within normal ranges. d-dimer was elevated at 5.47 µg/mL, and baseline hematocrit was 28.9%. Antithrombin III activity and fibrinogen were within normal ranges. Because of marked systemic and pulmonary congestion, intravenous furosemide was administered at doses of 40 mg, 20 mg, and 40 mg during the first 2 hospital days. Despite these relatively modest doses, the patient exhibited a marked diuretic response, resulting in a cumulative negative fluid balance of approximately 12 L over 48 hours (day 1, −8 L; day 2, −4 L), with substantial radiographic improvement of pulmonary congestion. The large negative fluid balance was not an intended therapeutic target, and no overt clinical signs of dehydration or hemodynamic instability requiring fluid resuscitation were observed during this period. However, on hospital day 3, her oxygen requirement increased despite improvement in pulmonary congestion. Chest radiography showed a new unilateral pulmonary opacity with pleural effusion, and hematocrit increased progressively to 33.9% by day 4, representing a 17.3% relative increase from baseline. The discordance between improved congestion and worsening hypoxemia prompted urgent computed tomography pulmonary angiography, which demonstrated extensive acute PTE involving the right main and segmental pulmonary arteries, with a large peripheral wedge-shaped consolidation compatible with pulmonary infarction (Fig. 2). At the time of PTE diagnosis, systolic blood pressure was approximately 90 mm Hg and heart rate was 123 beats/min. High-sensitivity cardiac troponin I was elevated at 0.074 ng/mL (reference range, 0–0.04 ng/mL). The simplified Pulmonary Embolism Severity Index was at least 3, and together with documented right ventricular systolic dysfunction (fractional area change, 27%) and elevated cardiac troponin, the patient was classified as having intermediate-high-risk PTE. Given the worsening hypoxemia, borderline hypotension, extensive thrombus burden, and limited cardiopulmonary reserve due to severe biventricular dysfunction, systemic thrombolysis was performed because of concern for further hemodynamic deterioration. The patient weighed approximately 55 kg, and no major contraindications to thrombolytic therapy were identified. Alteplase 50 mg was administered intravenously over 2 hours, followed by therapeutic anticoagulation with unfractionated heparin and subsequent transition to rivaroxaban. Guideline-directed medical therapy for HFrEF was initiated and titrated as tolerated. Although hemodynamic status gradually stabilized, the infarcted lung parenchyma recovered slowly. Despite thrombolysis and anticoagulation, radiographic improvement of the pulmonary infarction was minimal during the early phase. Chest radiography showed only partial improvement at 1 month, whereas complete radiographic resolution was achieved at 2 months (Fig. 3). At the 2-month follow-up, dyspnea had markedly improved, and serial echocardiography demonstrated improvement in LVEF to 37% with a progressive reduction in left ventricular size (Video S2, Supplemental Digital Content 2).

**Figure 1.**
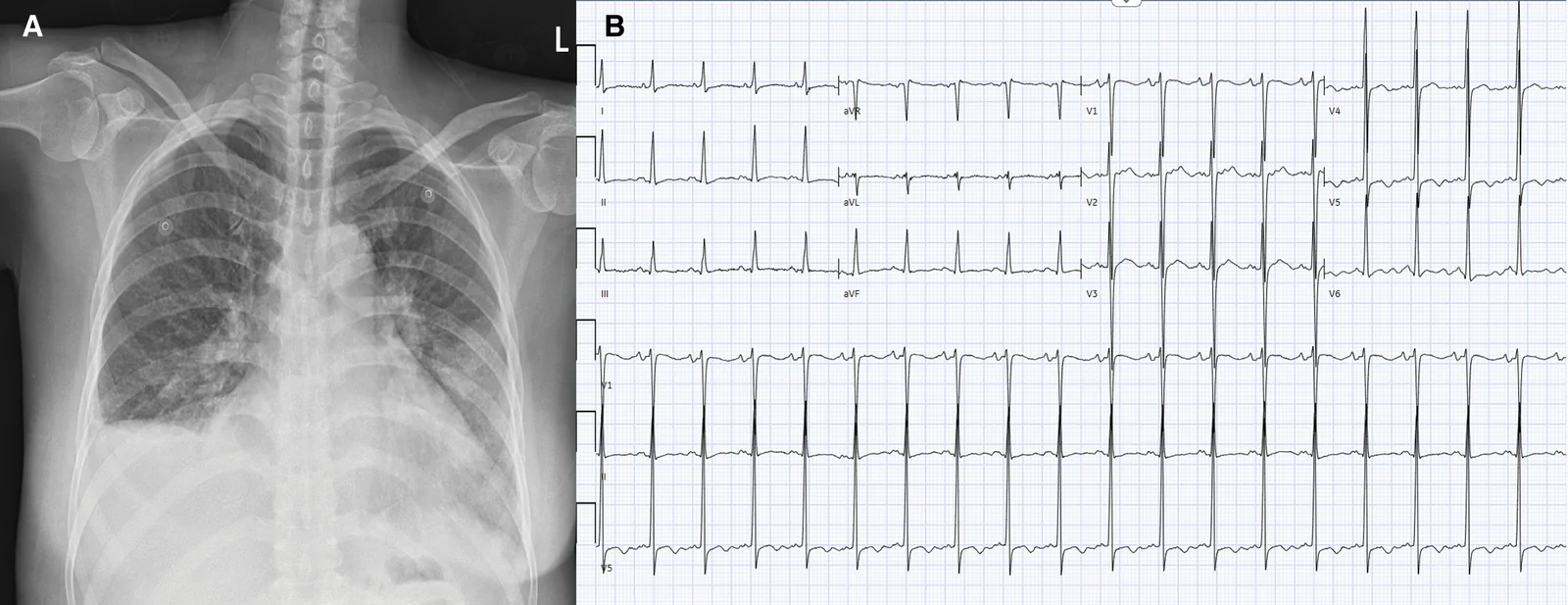
Admission findings. (A) Chest radiography showed bilateral pulmonary edema and pleural effusions. (B) Twelve-lead electrocardiography showed sinus tachycardia without ischemic changes or definite right heart strain.

**Figure 2.**
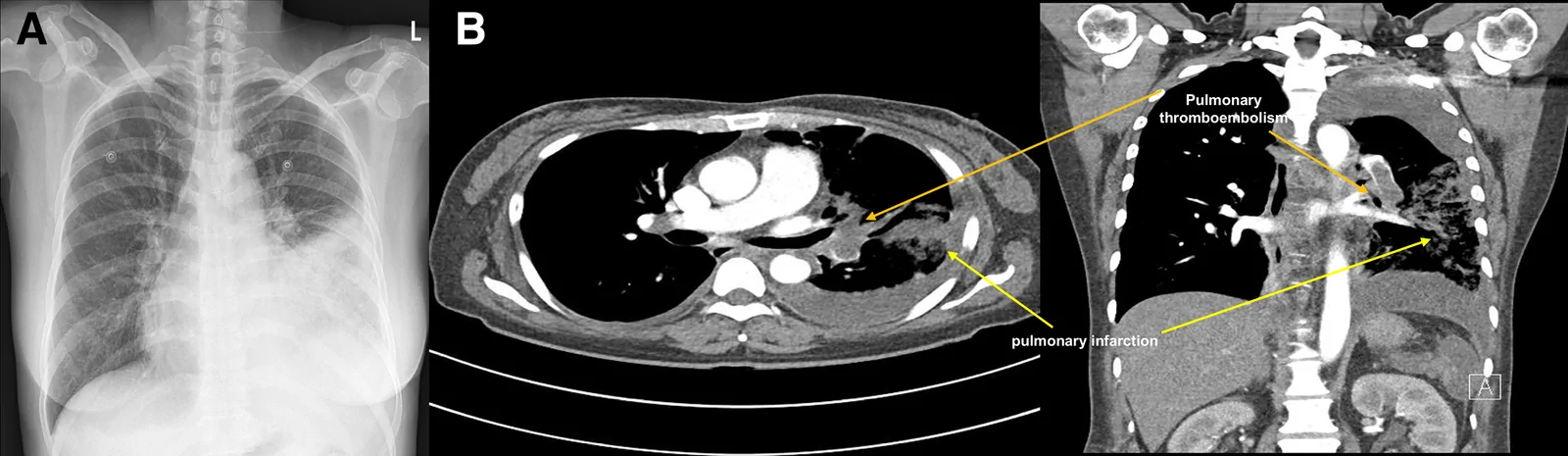
Acute pulmonary thromboembolism and pulmonary infarction after intensive decongestion. (A) Chest radiography on hospital day 3 showed a newly developed unilateral pulmonary opacity with pleural effusion despite improvement of pulmonary congestion. (B) Computed tomography pulmonary angiography demonstrated filling defects in the pulmonary arteries and a large peripheral wedge-shaped consolidation compatible with pulmonary infarction.

**Figure 3.**
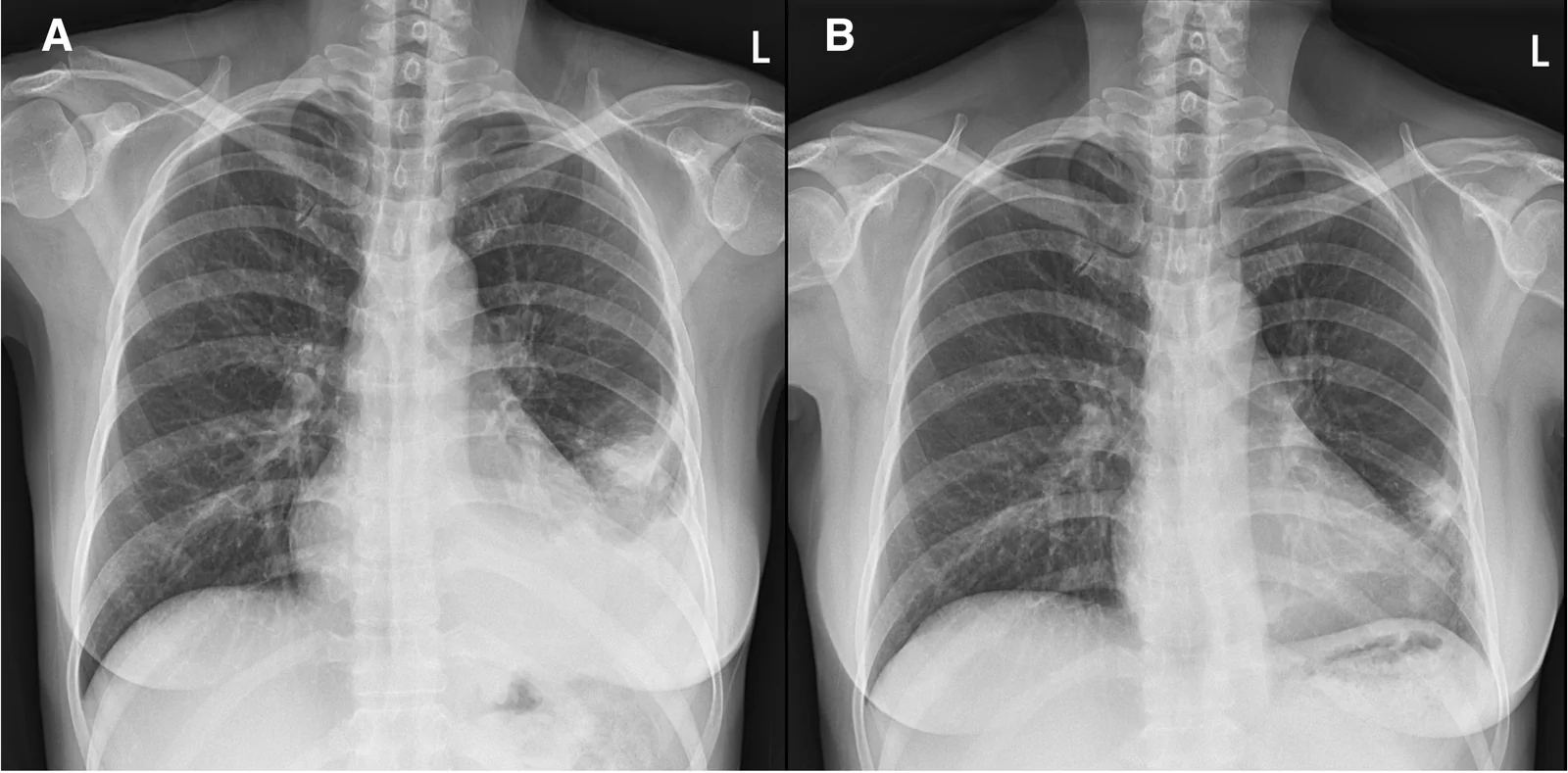
Serial follow-up chest radiography. (A) At 1 month, the pulmonary infarction showed partial radiographic improvement. (B) At 2 months, the pulmonary opacity and pleural effusion had nearly completely resolved.

## 3. Discussion

This case highlights the diagnostic challenge of PTE in acute decompensated heart failure and the potential for delayed radiographic recovery of pulmonary infarction. Heart failure is associated with an increased risk of venous thromboembolism, particularly during hospitalization.^[1-4]^ Reduced cardiac output, venous stasis, endothelial dysfunction, neurohormonal activation, and systemic inflammation may collectively create a prothrombotic milieu. In this patient, relatively modest doses of intravenous furosemide produced an unexpectedly marked diuretic response, resulting in a substantial negative fluid balance and progressive hemoconcentration. Although adequate decongestion is essential in acute heart failure, the intensity of diuretic therapy should be individualized according to the degree of congestion and diuretic response. Close monitoring of volume status, hemodynamics, renal function, and hemoconcentration is important to avoid excessive intravascular volume depletion during decongestive therapy.^[7,8]^ Thus, rapid volume removal may have transiently intensified an already prothrombotic state in the setting of severely reduced left ventricular systolic function. However, because d-dimer was already elevated at admission and lower-extremity venous ultrasonography was not performed, a direct causal relationship between diuresis and PTE cannot be established. The diagnosis of PTE in decompensated heart failure is challenging because dyspnea, hypoxemia, tachycardia, pleural effusion, and radiographic pulmonary abnormalities can occur in both conditions.^[2,5]^ In this case, the key diagnostic clue was the discordance between improvement in pulmonary congestion and worsening oxygenation, accompanied by a newly developed right-sided opacity. This clinical trajectory was inconsistent with persistent heart failure alone and prompted computed tomography pulmonary angiography. In complex hospitalized patients, clinical suspicion based on the overall trajectory remains essential because D-dimer alone is insufficient to distinguish PTE from other acute conditions.^[5]^

Thrombophilia evaluation was limited to antithrombin III activity and fibrinogen, both of which were within normal limits. These findings do not exclude other inherited or acquired thrombophilic disorders. Current guidance recommends selective rather than routine thrombophilia testing after venous thromboembolism, particularly when results may affect anticoagulation duration, family counseling, or evaluation of recurrent or unusual-site thrombosis.^[9]^ Therefore, the absence of a comprehensive thrombophilia panel should be acknowledged as a limitation.

A notable feature of this case is the delayed resolution of pulmonary infarction. Pulmonary infarction reflects ischemia, hemorrhage, and necrosis of lung parenchyma distal to pulmonary arterial obstruction.^[10-12]^ Unlike thromboembolic obstruction, infarcted parenchyma requires gradual resorption of hemorrhage and resolution of inflammatory change.^[12,13]^ In this patient, pulmonary infarction resolved slowly despite prompt thrombolysis and anticoagulation, with partial radiographic improvement at 1 month and complete resolution at 2 months. Persistent pulmonary opacity after PTE should therefore be interpreted together with symptoms, oxygenation, and evidence of recurrent thromboembolism, rather than being considered treatment failure based on imaging alone.

The improvement in left ventricular ejection fraction from 25% to 37%, with reduction in left ventricular size, was likely multifactorial. Guideline-directed medical therapy can induce reverse remodeling in newly diagnosed nonischemic dilated cardiomyopathy.^[6,14]^ In addition, resolution of extensive PTE may reduce right ventricular pressure overload and improve left ventricular filling through ventricular interdependence.^[15]^ However, the relative contribution of heart failure therapy and PTE resolution cannot be determined in a single case.

## 4. Conclusion

In patients with severe heart failure, decongestive therapy should be individualized with careful monitoring to avoid excessive intravascular volume depletion, and PTE should be promptly considered when respiratory status unexpectedly worsens despite improvement in pulmonary congestion. Even after appropriate treatment, pulmonary infarction may show delayed radiographic resolution over several weeks to months.

## Acknowledgments

The patient had provided informed consent for the publication of his clinical data and associated images for academic purposes.

## Author contributions

**Conceptualization:** Jong-Il Park.

**Data curation:** A-Reum Roh, Jong-Il Park.

**Investigation:** A-Reum Roh, Jong Hyun Baek, Jong-Il Park.

**Supervision:** Jong-Il Park.

**Writing – original draft:** A-Reum Roh, Jong-Il Park.

**Writing – review & editing:** A-Reum Roh, Jong-Il Park.




